# Nuclear halo measurements for accurate prediction of field size factor in a Varian ProBeam proton PBS system

**DOI:** 10.1002/acm2.12783

**Published:** 2019-12-02

**Authors:** Joseph Harms, Chih‐Wei Chang, Rongxiao Zhang, Liyong Lin

**Affiliations:** ^1^ Department of Radiation Oncology Emory University Atlanta GA USA; ^2^ Department of Radiation Oncology Dartmouth University Hanover NH USA

**Keywords:** experimental dosimetry, halo, Monte Carlo, PBS, proton beam commissioning, proton therapy

## Abstract

**Purpose:**

For pencil‐beam scanning proton therapy systems, in‐air non‐Gaussian halo can significantly impact output at small field sizes and low energies. Since the low‐intensity tail of spot profile (halo) is not necessarily modeled in treatment planning systems (TPSs), this can potentially lead to significant differences in patient dose distribution. In this work, we report such impact for a Varian ProBeam system.

**Methods:**

We use a pair magnification technique to measure two‐dimensional (2D) spot profiles of protons from 70 to 242 MeV at the treatment isocenter and 30 cm upstream of the isocenter. Measurements are made with both Gafchromic film and a scintillator detector coupled to a CCD camera (IBA Lynx). Spot profiles are measured down to 0.01% of their maximum intensity. Field size factors (FSFs) are compared among calculation using measured 2D profiles, calculation using a clinical treatment planning algorithm (Raystation 8A clinical Monte Carlo), and a CC04 small‐volume ion chamber. FSFs were measured for square fields of proton energies ranging from 70 to 242 MeV.

**Results:**

All film and Lynx measurements agree within 1 mm for full width at half maximum beam intensity. The measured radial spot profiles disagree with simple Gaussian approximations, which are used for modeling in the TPS. FSF measurements show the magnitude of disagreements between beam output in reality and in the TPS without modeling halo. We found that the clinical TPS overestimated output by as much as 6% for small field sizes of 2 cm at the lowest energy of 70 MeV while the film and Lynx measurements agreed within 4% and 1%, respectively, for this FSF.

**Conclusions:**

If the in‐air halo for low‐energy proton beams is not fully modeled by the TPS, this could potentially lead to under‐dosing small, shallow treatment volumes in PBS treatment plans.

## 1 INTRODUCTION

Pencil‐beam scanning (PBS) proton therapy is rapidly expanding throughout the United States and the world because of its superiority to both x‐ray and passive‐scattering proton therapies in terms of dose conformality and normal tissue sparing. As PBS allows for intensity‐modulated proton therapy (IMPT), the importance of the beam modeling accuracy is increased relative to photon therapies because target volumes are treated with more PBS spots than IMRT MLC segments. Commissioning of a treatment planning system (TPS) for clinical use with a PBS system involves measurements of integrated depth‐dose curves (IDDs), lateral beam spot profiles, and absolute outputs. As PBS treatment is composed of superposition of many proton beam spots, an integral part of commissioning clinical PBS systems is evaluation of the broad tails of spot profiles. In early model‐based dose calculations, dose deposition was modeled as the convolution of the in‐air fluence with a dose kernel. The dose kernel is a three‐dimensional (3D) in‐water dose distribution of an infinitesimal proton beam and consists of an IDD and a lateral distribution (i.e., a spot profile). This simplified approach in the TPS led to dose errors of up to 15% at depth because it relies on a Gaussian approximation, either single or double Gaussian, to model the lateral spot profile.^1^ This simplified model neglects the low‐dose envelope surrounding each spot. This envelope, or nuclear halo, is created by large angle scattering in beam line components for low‐energy beams and secondary particles produced in the medium for high‐energy beams.^2^, ^3^, ^4^ For individual spots, this effect may be minimal, but in IMPT, when the beam is scanned three dimensions over a large volume, the cumulative effect of this halo can become significant especially for treatment delivery systems that have smaller spots as the number of spots needed for the same treatment site typically increase with smaller spots. Zhu et al reported that output for small fields could have up to 13% disagreement between TPS and measurement for a single‐Gaussian model, and up to 7.2% disagreement when a double Gaussian model was used.^5^

For high energies, when the nuclear halo is depth dependent, this effect has been studied and characterized with Monte Carlo‐based dose calculation algorithms.^6^ With more widespread implementation of clinical Monte Carlo algorithms, and enhanced corrections for model‐based algorithms, the halo effect at depth can be modeled within 1–2% accuracy for high‐energy beams.^5^, ^7^, ^8^ However, Monte Carlo algorithms do not model the halo that is created upstream from the treatment volume, for example, in a range shifter.^9^ This can lead to discrepancies for low‐energy proton beams as the halo is generated in both the treatment head and through scattering in air.^10^ In commissioning our Varian ProBeam PBS system, we found the largest disagreement between TPS and measurement was 8% for a 2 × 2 cm^2^ monoenergetic field at 70 MeV. Several authors have previously measured the halo effect on various treatment delivery systems. As mentioned above, the low‐energy halo is due to scattering in the treatment head and in air, so the halo effect will vary between machine designs. Lin et al developed a measurement technique to measure the halo effect by measuring spot profiles down to very low intensities. These measurements have previously been reported for an IBA PBS nozzle.^11^ Wurl et al used ion chambers for measurement and validated a FLUKA Monte Carlo (MC) simulation to model the halo effect for a Varian ProBeam system.^10^ The results obtained by Wurl et al were used to influence analytical dose calculations. While the authors found that their MC simulation was able to predict the halo effect, we have found that our clinical MC algorithm, RayMC 8A using single‐Gaussian model, could not accurately predict the output, especially for low energies and small fields. The proposed study differs from the previous study in that two‐dimensional (2D) proton spot profiles were measured for validation of a clinical MC‐based treatment planning system.

## 2 MATERIALS AND METHODS

### 2.1 Pair magnification

Spot profiles can be measured in a variety of ways, including scanning ion chambers,^7^ ion chamber arrays,^3^ radiochromic films,^13^ and CCD‐coupled scintillation screens.^7^, ^12^, ^13^ Film and scintillator systems offer the convenience of simultaneous measurements of the 2D spot profile, greatly enhancing efficiency and reducing the amount of beam‐on time required for commissioning a PBS system, relative to scanning ion chambers. However, a limitation of both media is their dynamic range. The IBA Lynx scintillator‐CCD system has a range from 0 to 1000 counts, which limits its ability to measure low‐dose tails for spot profiles with reasonable accuracy. Additionally, Gafchromic EBT3 film is also limited for its use in low‐dose tail measurements as its useful range is 0.01–8 Gy. To overcome these limitations, we employed the pair/magnification technique proposed by Lin et al.^14^ With this approach, the full spot profile can be measured with as low as 0.2% uncertainty for relative intensities >0.01% of the central beam intensity. In this work, we employ this pair magnification technique using film and the Lynx detector to measure the halo effect for a Varian ProBeam PBS system. These spot measurements are then used to build a simple simulation to calculate relative field size factors (FSFs) as a function of energy, and these are compared to measurements of FSFs which were taken during commissioning.

As the pair magnification method has been previously discussed in detail, here we provide a summary of the method, and refer the reader to the various publications by Lin et al for further details of the method.^14^ The goal of this method is to increase the dynamic range of the radiation detector (either film or a scintillator‐CCD pair) by pairing successive irradiations at increasing dose. Using film as an example, the first irradiation will have a maximum dose of around 6 Gy ensuring that no portion of the film is saturated. Successive irradiations then will magnify the output, delivering, for example, 20 times as much dose in the first magnification, and 400 times as much dose in the second magnification. After measurements the film is scanned and spatially registered to form one composite image. The same process is applied to the Lynx data. This process will allow for measurements with 0.2% relative uncertainty at signal levels as low of 0.01% of the central axis intensity.

In this work, the pair magnification technique was carried out for monoenergetic proton beams at 70, 80, 90, 100, 130, 150, 180, 210, and 242 MeV. For each energy, measurements were taken at the treatment isocenter and at 30 cm upstream from isocenter (i.e., near the treatment snout). As mentioned above, measurements were taken with Gafchromic EBT3 film and an IBA Lynx detector for independent verification.

### 2.2 Field size factor measurements

As part of the commissioning process for our ProBeam system, FSFs were measured for monoenergetic proton beams at 70, 100, 130, 150, 180, 210, and 242 MeV in a SAD setup. The square fields used a 2.5 mm spot spacing (for all energies) and side lengths of 2, 3.2, 6, 10, 14, and 20 cm. A small‐volume CC04 ion chamber of 4 mm diameter and 3.6 mm length (IBA Dosimetry, Bartlett, TN) was placed at 0.62 cm depth in a water tank at the center of the field. The depth was chosen to match the water equivalent thickness of the IBA MatriXXPT.^15^ All FSFs are normalized to the dose measured for the 10 cm field per each energy.

To model the FSF using the halo measurements, the spot profile was converted into a matrix and superimposed over a square field space. Raystation models the spot spacing as a function of treatment depth.^16^ Since these plans were calculated in air at isocenter, the center‐to‐center spot spacing is 2.5 mm for every energy layer. The simulation implemented spot positions and spot scanning as in the TPS. The same field sizes used for measurement were used for simulation. This was repeated for each field size for both film‐ and Lynx‐derived datasets.

## 3 RESULTS

### 3.1 Spot profiles

**Figure 1:**
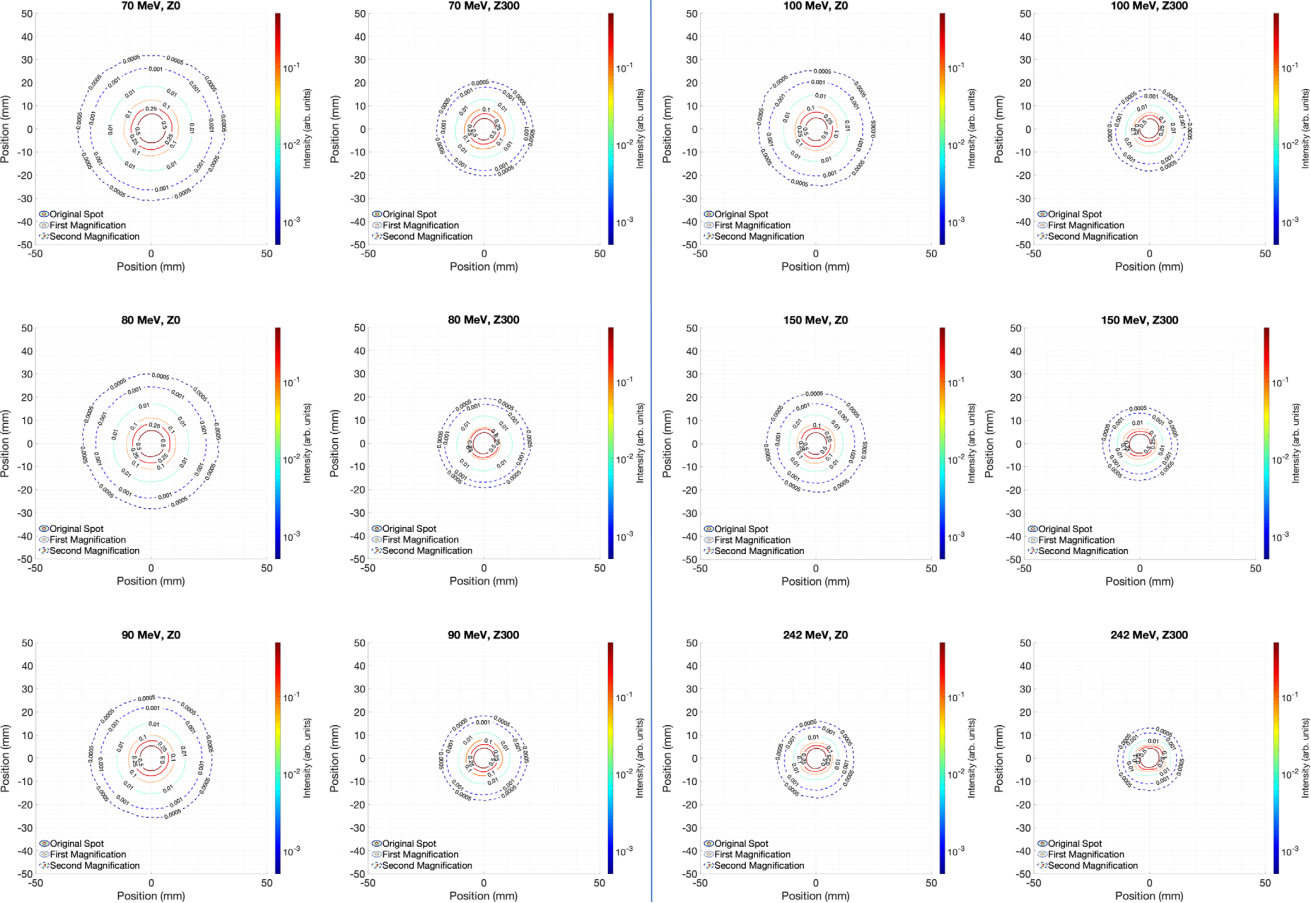
Film‐derived spot profiles using the pair magnification technique. Z0 corresponds to measurements taken at the isocenter and Z300 corresponds to measurements taken 300 mm upstream.

**Figure 2:**
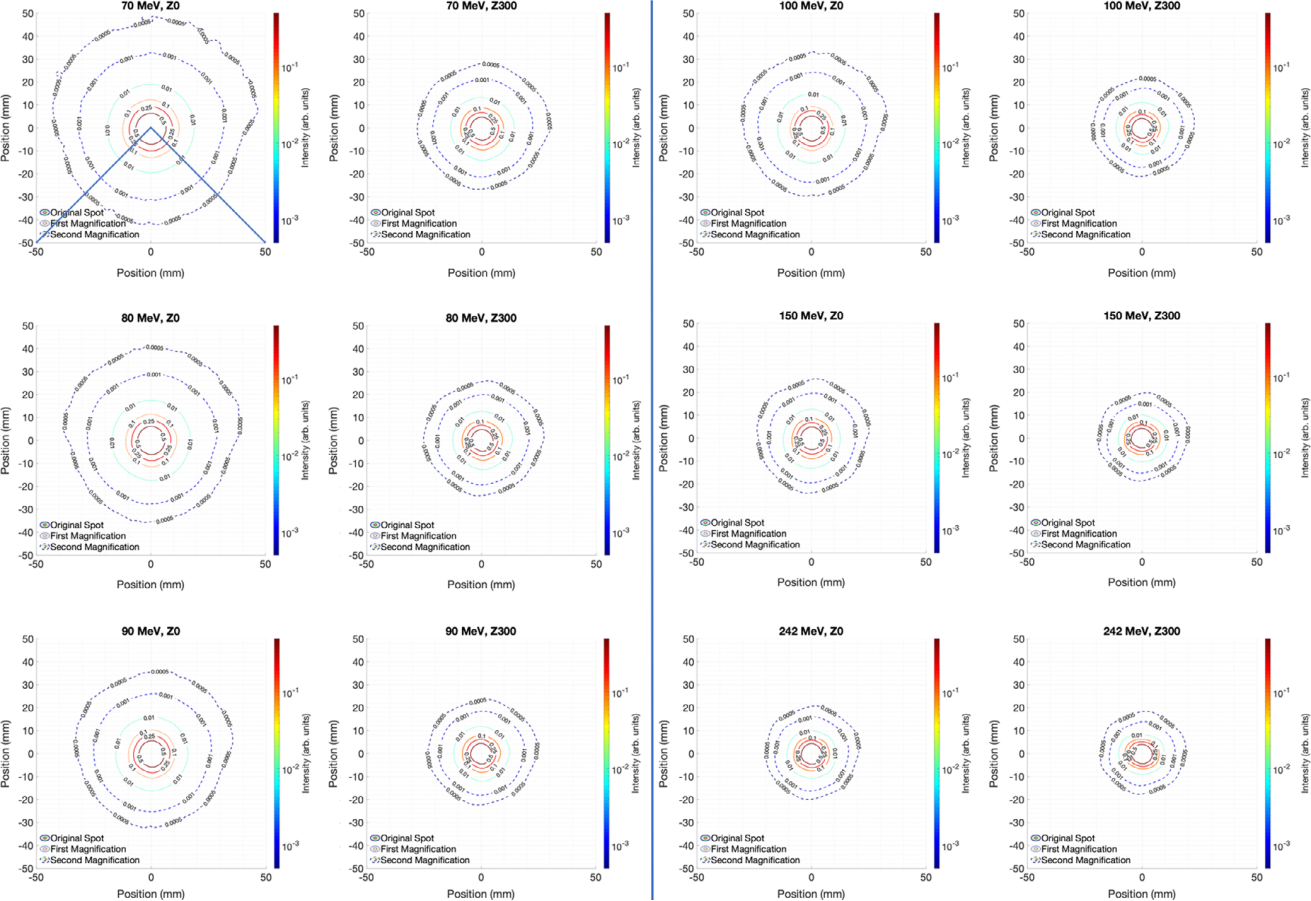
Lynx‐derived spot profiles using the pair magnification technique. The Lynx acquisition system showed an over‐response artifact in the positive y direction on these figures. The area enclosed by the blue lines on the 70 MeV, Z0 spot profile is used for calculation of one‐dimensional line profiles.

The spot profile measurements are summarized in Fig. 1 for the film data and Fig. 2 for the Lynx data. Limited data are shown for brevity. Using both measurement devices, the spot profiles are noticeably larger at isocenter than near the snout. This effect is especially pronounced at 70–90 MeV.

Figure 2 shows the results of the Lynx measurements, which have an over‐response artifact. The lower portions of the figure (negative on the *y*‐axis) correspond to the true halo while the upper portions are degraded by the effect, which was as high as a 40% overestimation at low dose. Such overestimation can be avoided by setting the beam entrance away from the CCD camera in the Lynx neck and assessed by comparing off center acquisition to that at the center. Another approach used for correcting this artifact is presented below in the discussion of radial spot profiles. Aside from the artifact, the Lynx results appear to be consistent with the film results qualitatively. Both sets of measurements show that the growth in spot size from the snout to the isocenter is larger for the lower energy protons as we would expect.

**Figure 3:**
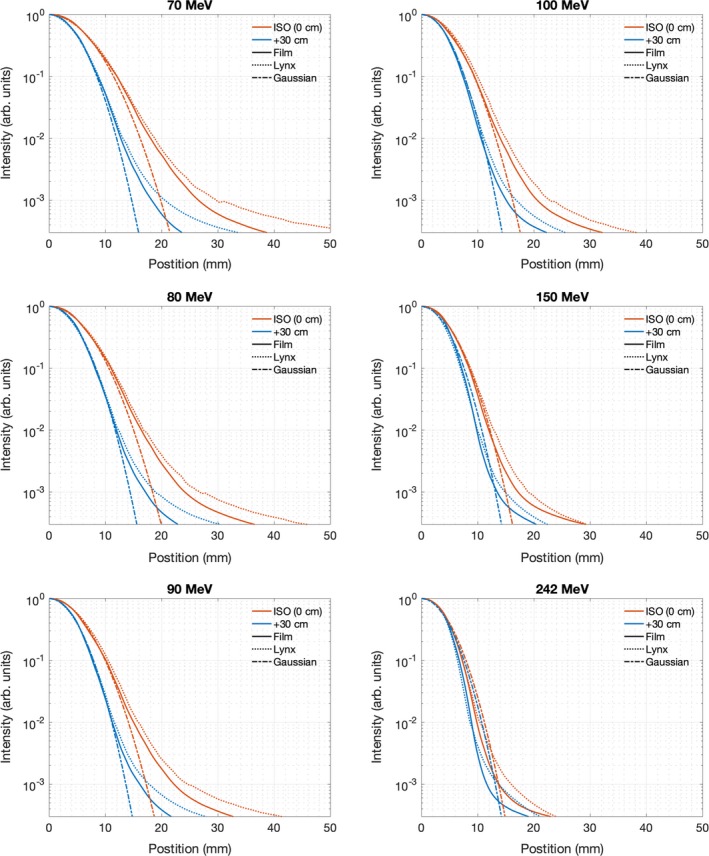
Radially averaged spot profiles, three pairs of curves for the film, Lynx, and Gaussian fit data at two in‐air locations. Gaussian profiles based on the average sigma from the measurements are shown for comparison. The color of each line is meant only to show the position (0 or + 30 cm) where each measurement was taken, and the line pattern (solid, dotted, dashed) shows if film, Lynx, or Gaussian fit was used.

In order to provide a more quantitative comparison between the measurements, Fig. 3 shows radially averaged line profiles along with Gaussian profiles based on the average sigma from the measurements. For the measured profiles, the data were converted to polar coordinates, with the maximum intensity of each measurement corresponding to the origin. The data are then averaged over each polar angle as a function of radius from the center. Averaging the data in this way further reduces effects of noise which may be present in Figs. 1 and 2. Because of the Lynx artifact, only the spot data extending from polar angles 135–225 (the quarter of the Lynx that is far from the camera neck, enclosed by blue lines on Fig. 2) are included in the radial profile. While the data disagree below 0.1% maximum intensity, they are consistent above this point. Tables 1, 2, 3 show the full width at half maximum (FWHM), full width at 10% maximum, and full width at 1% maximum for each proton energy measured. As with Fig. 3 the images are largely consistent between Lynx and film; the largest error for the FWHM data is 1.0 mm, which is a difference in spot sigma of 0.4 mm. The largest discrepancy, at 100 MeV, is likely due to a combination of both measurement uncertainty of 0.5 mm Lynx resolution and machine spot size variation at Varian tolerance of 15%. This error lies within that both Lynx resolution and Varian tolerance. The average disagreement between film and Lynx for FWHM and FW10% is <5% at both measurement positions, while the disagreement goes up to 6% for FW1% for the measurements taken at isocenter.

**Table 1:** Full width at half maximum for the radially averaged spot profiles in mm.

Energy	Z = 0				Z = 300			
	Film	Lynx	Raw diff.	Percent diff.	Film	Lynx	Raw diff.	Percent diff.
70	12.5	12.6	−0.1	−0.9%	9.4	9.2	0.2	2.6%
80	11.5	11.8	−0.3	−2.3%	9.4	8.9	0.5	5.4%
90	10.7	11.2	−0.5	−4.6%	8.8	8.5	0.3	2.9%
100	9.8	10.7	−1.0	−9.4%	8.3	8.4	−0.1	−1.5%
130	9.5	9.9	−0.4	−3.9%	8.5	8.1	0.4	4.6%
150	9.4	9.5	0.0	−0.4%	8.8	7.9	0.9	10.6%
180	9.1	9.2	−0.2	−1.9%	8.8	8.3	0.5	6.2%
210	9.0	8.7	0.3	3.6%	8.7	7.9	0.8	9.2%
242	8.7	8.7	0.1	0.7%	8.4	8.2	0.2	2.2%
Mean			−0.2	−2.1%			0.4	4.7%

**Table 2:** Full width at 10% of the maximum for the radially averaged spot profiles in mm.

Energy	Z = 0				Z = 300			
	Film	Lynx	Raw diff.	Percent diff.	Film	Lynx	Raw diff.	Percent diff.
70	24.1	24.2	−0.1	−0.6%	17.3	17.5	−0.2	−1.3%
80	22.0	22.3	−0.3	−1.5%	16.3	16.5	−0.3	−1.6%
90	20.2	21.0	−0.8	−3.9%	15.3	15.8	−0.4	−2.7%
100	18.8	19.9	−1.1	−5.5%	14.7	15.3	−0.6	−3.8%
130	17.4	18.2	−0.8	−4.4%	14.0	14.5	−0.5	−3.5%
150	16.9	17.5	−0.6	−3.6%	14.3	14.0	0.2	1.7%
180	15.9	16.5	−0.7	−4.1%	14.0	13.5	0.4	3.3%
210	15.2	15.5	−0.4	−2.4%	13.8	13.0	0.8	6.1%
242	14.5	14.5	0.0	−0.1%	13.4	13.0	0.4	2.9%
Mean			−0.5	−2.9%			0.0	0.1%

**Table 3:** Full width at 1% of the maximum for the radially averaged spot profiles in mm.

Energy	Z = 0				Z = 300			
	Film	Lynx	Raw diff.	Percent diff.	Film	Lynx	Raw diff.	Percent diff.
70	36.1	37.6	−1.5	−4.1%	25.5	26.2	−0.8	−2.9%
80	32.8	33.6	−0.8	−2.4%	23.9	24.7	−0.7	−3.0%
90	30.0	31.7	−1.7	−5.5%	22.5	23.2	−0.7	−3.1%
100	28.0	30.0	−1.9	−6.7%	21.2	22.3	−1.1	−5.1%
130	25.1	26.9	−1.7	−6.7%	19.8	20.7	−1.0	−4.9%
150	23.9	25.8	−1.8	−7.3%	19.6	20.0	−0.4	−2.0%
180	22.2	24.0	−1.9	−8.1%	19.0	19.3	−0.3	−1.4%
210	20.9	22.4	−1.6	−7.2%	18.5	18.3	0.2	1.2%
242	19.7	20.8	−1.1	−5.6%	17.8	17.3	0.5	2.9%
Mean			−1.6	−6.0%			−0.5	−2.0%

### 3.2 Field size factor comparison

**Figure 4:**
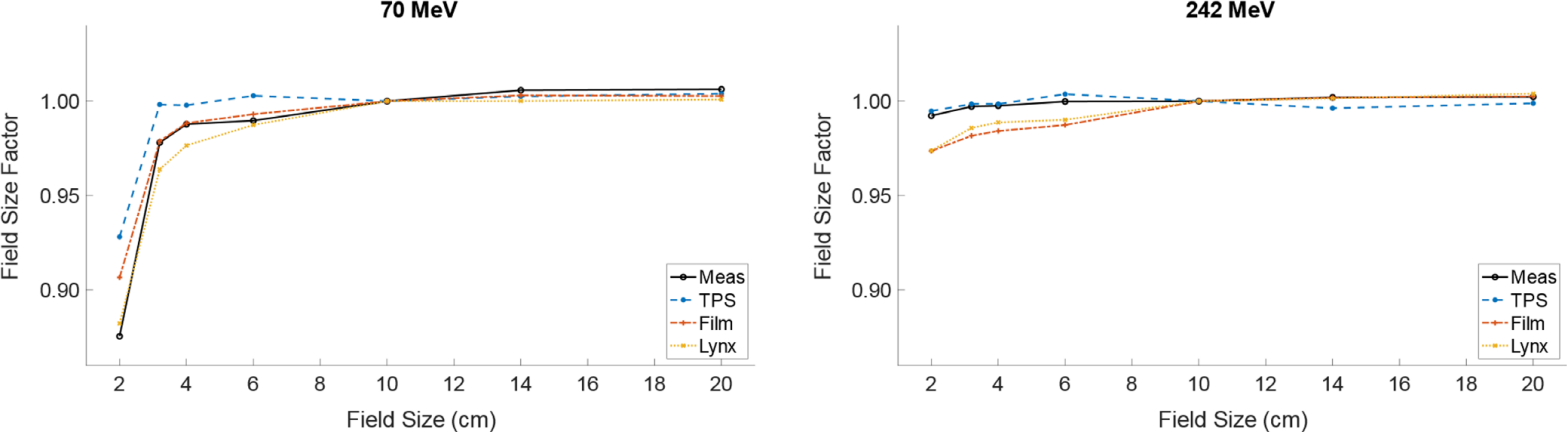
Field size factors for the lowest and highest energies available on our machine. The data labeled “Meas” was measured with a CC04 ion chamber. The film and Lynx values come from a superposition of spot measurements over a grid corresponding to the appropriate field size and spot spacing. All film and Lynx measurements are taken at the surface and ion chamber measurements are taken at a depth of 0.62 cm.

Field size factors display the aggregate effects of incorrect spot modeling for relatively simple plans. Figure 4 shows the FSFs at 70 and 242 MeV. At low energies and small field sizes, the TPS does not accurately predict the FSF, using the ion chamber measurements as a reference. To account for volume averaging effects of the chamber, a sphere with radius equal to the chamber (around 2 mm) was used in addition to point doses in the TPS. It was found that the volume average does not change the dose by more than 0.3%. The film data and Lynx more accurately predict this value using the calculation based on the spot profile including halo. Table 4 shows the error for each FSF measurement for all field sizes at 70 MeV, relative to the ion chamber measurements.

**Table 4:** Percent error (relative to ion chamber measurements) for all FSFs at 70 MeV.

Field size (cm)	TPS	Film	Lynx
2.0	6.0%	3.6%	0.8%
3.2	2.1%	0.1%	−1.5%
4.0	1.0%	0.1%	−1.1%
6.0	1.3%	0.3%	−0.2%
10.0	0.0%	0.0%	0.0%
14.0	−0.3%	−0.3%	−0.6%
20.0	−0.2%	−0.4%	−0.5%
Mean	1.4%	0.5%	−0.5%

## 4 DISCUSSION

The results presented in this work show that the Lynx detector should be used with caution for in‐air halo measurements. This also highlights the need for independent measurement to verify which region of these data (toward or away from the camera neck) was accurate for measurement.

At 1% of the maximum signal, the full spot width is over 36 mm for a 70 MeV proton spot, which exemplifies the importance of including halo data in the beam model. In Raystation 8A's clinical Monte Carlo algorithm, each spot is defined by a Gaussian distribution, leading to predicted spot sizes of 22.6 mm at 1% intensity. This means the TPS would predict almost no dose beyond 22.6 mm when there is dose being delivered up to 36 mm from the center of the spot, as confirmed by these measurements. While previous work has shown that Monte Carlo algorithms effectively model the nuclear halo at depth for high energy proton beams, this work shows that output at superficial depths for small fields is poorly modeled by the commercial TPS because the in‐air nuclear halo is ignored. This is because the halo for low‐energy beams is created in the treatment head rather than nuclear events in medium. As mentioned above, previous investigators have shown that a double Gaussian can be used to model the nuclear halo effect both in air and in medium.^17^ Shen et al and Lin et al both showed that inclusion of a double Gaussian spot profile can more accurately model FSF even for Monte Carlo dose calculation.^18^, ^19^ Based on the results presented in this work, in addition to the work done by Shen et al, inclusion of this double Gaussian in the commercial TPS could help mitigate the presented disagreements.

In clinical practice, treatment fields are delivered with spread‐out Bragg peaks, and the lowest energy fields, where the halo effect is most prominent, contribute relatively little weight to the overall treatment field. However, this work suggests the simple Gaussian spot profile approximation used in the TPS could under‐predict skin dose for these small‐weighted contributions for deep seated small breast cancer treatment plans without range shifter. The expected clinical impact could be relative underestimation of dose toward the surface for these low‐energy layers. In the work by Shen et al, the authors showed that inclusion of double Gaussians in the beam model improved gamma passing rates for head‐and‐neck treatment plans. Even though Shen et al found that inclusion of a double Gaussian in Eclipse predominantly helped the FSF for large fields, we believe that inclusion of a double Gaussian could help the model for small field FSFs. This is because the in‐air halo extension in ProBeam is smaller at radii from 2 to 5 cm than that of the in‐medium halo, at radii up to 10 cm. Further investigation of this effect could include surface dose measurements for clinical treatments plans using OSLDs. Additionally, many shallow treatment fields are treated with range shifters, which are expected to exacerbate the halo effect. Future investigations could include measurement of FSFs with range shifters in order to more accurately quantify these discrepancies over independent dose calculations outside of TPS.^18^

## 5 CONCLUSIONS

In this work, we have shown the magnitude of disagreement over output of small fields without modeling halo for a Varian Probeam system. These measurements are taken with both Gafchromic film and a scintillating detector optically coupled to a CCD using the previously published pair magnification technique. This technique allows for measurements down to 0.01% of the signal intensity with 0.2% uncertainty. The spot profiles are used for a simple simulation to compare to FSF measurements taken with a CC04 ion chamber. These data are compared to values predicted by Raystation 8A's treatment planning system, which predicted a higher FSF than was measured with the ion chamber. Film and Lynx data agree within 3.6% and 1.5%, respectively, of the ion chamber measurements for small field sizes (≤4 cm) at 70. The TPS modeled values with 6.0% agreement.

## CONFLICT OF INTEREST

The authors have no conflict of interest.
